# Cell-Penetrating Fragments of the Cdk5 Regulatory Subunit Are Protective in Models of Neurodegeneration

**DOI:** 10.3390/ph3041232

**Published:** 2010-04-23

**Authors:** Jan Liman, Jochen H. Weishaupt, Mathias Bähr, Gunnar P.H. Dietz

**Affiliations:** 1 Department of Neurology, University of Göttingen, Robert-Koch Str. 40 37075 Göttingen, Germany; 2 DFG-Research Center for Molecular Physiology of the Brain (CMPB), Humboldtallee 23, 37075 Göttingen, Germany; E-Mails: jliman@gwdg.de (J.L.); jochen.weishaupt@medizin.uni-goettingen.de (J.H.W.); mbahr@gwdg.de (M.B.); 3 Department 828, Molecular Neurobiology, H. Lundbeck A/S, Ottiliavej 9, 2500 Valby, Denmark

**Keywords:** Cdk5, Tat-technique, neurodegenerative diseases

## Abstract

Cdk5 is essential for neuronal differentiation processes in the brain. Activation of Cdk5 requires the association with the mostly neuron-specific p35 or p39. Overactivation of CDK5 by cleavage of p35 into p25 is thought to be involved in neurodegenerative processes. Here, we have tested an approach to inhibit pathological Cdk5 activation with a Tat-linked dominant-negative fragment of p25. It reduced cell death induced by staurosporine and showed a tendency to alleviate manganese-induced cell death, while it did not protect against 6-OHDA toxicity. Our results suggest that the Tat technique is a suitable tool to inhibit dysregulated CDK5.

## 1. Introduction

Cdk5 belongs to the family of cyclin-dependent kinases, which are essential for cell cycle and differentiation processes. In contrast to other Cdks, Cdk5 does not require the association with a specific cyclin, but the neuron specific protein p35 for activation. This Cdk5/p35 complex is essential for neurite outgrowth, cortical development, and other differentiation processes [1]. Cellular stress can lead to a calcium influx into the cell with consecutive activation of calpain, which cleaves p35 into p25, resulting in an aberrant activation of Cdk5. This mechanism is thought to be involved in neurodegenerative diseases such as Parkinson’s disease, Amyotrophic Lateral Sclerosis or Alzheimer’s dementia [2,3,4]. In post mortem brains of Alzheimer’s disease patients, enhanced processing of p35 to the p25 fragment, resulting in an increased p25/35 ratio, has been found [5]. p25/Cdk5 can also induce intraneuronal accumulation of beta Amyloid *in vitro* and *vivo* [6]. Moreover, it has been shown that pathological activation of Cdk5 by p25 can lead to hyperphosphorylation of Tau in various models [7]. Vice versa it has been shown that inhibition of p25 formation can abolish Tau hyperphosphorylation [8]. The cleavage of p35 to p25 is also found in models for Parkinson’s disease and Huntington’s disease, underlining its general importance as a pathological process in neurodegenerative diseases [9]. p25 is cleaved by calpain after different apoptotic stimuli [10]. To date, different approaches have been made to inhibit pathological Cdk5 activation, e.g. Cdk5 inhibition by the small molecule pan-CDK inhibitor roscovitine, which did not block pathological Tau phosphorylation [6], or application of a calpain inhibitor, which could decrease p25 levels and abrogate tau hyperphoshporylation [8]. In our study we have addressed a challenge of previous experiments, which is to specifically inhibit pathological Cdk5 activation, and to deliver such an inhibitor to its target site. In recent years, strategies have been developed to overcome the blood brain barrier and cellular membranes [11,12]. One approach is to link the protein of interest to basic peptides, such as the cell-penetrating peptide (CPP) derived from the HIV Tat protein [13]. Here, we have generated a Tat-linked dominant negative p25 fusion protein and tested it in cellular models for oxidative stress and apoptosis, as oxidative stress is thought to be one factor in the development of various neurodegenerative diseases.

## 2. Experimental Section

### 2.1. Cloning of constructs and purification of Tat fusion proteins

Tat-p25 constructs were generated from expression vectors in *E. coli* and purified via Ni-affinity chromatography as described [14,15]. S.F. Dowdy (San Diego, CA) kindly provided the pTAT-HA vector that we used to generate the Tat-p25 expression constructs [13]. 3 different p25 cDNA fragments were selected according to their previously described functions [16]. Amino acid residues 122–291 (N122) served as a positive control, residues 200–291 (N200) as a negative control, residues 150–279 (N279) as the dominant negative p25 fragment. 

**Figure 1:**
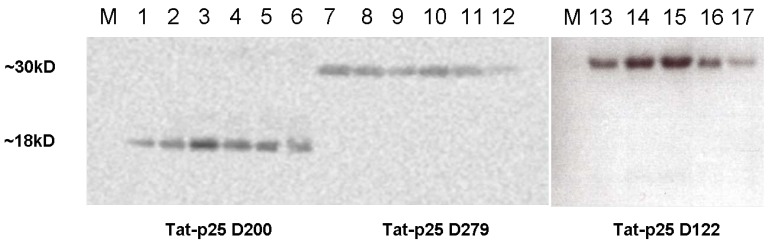
Integrity of fusion proteins confirmed by Western analysis. After purification of recombinant protein by nickel affinity chromatography, column fractions 4–9 (lane 1–6: Tat-p25 D 200; lane 7–12: Tat-p25 D279, lane 13–17: Tat-p25 D122) were analysed by western blotting using an antibody against the hemagglutinin tag included in the recombinant protein.

For expression of the p25 mutants, p25 full length cDNA was generated by RT PCR amplification from whole mouse brain using the primer GCTACAGGGGGC3′ (MWG Biotech AG Ebersberg, Germany) followed by PCR amplification with the primers AGTCATCCATGGTCAAGAAGGCCCC GCACCC/GTCATGAATTCATTCTTCAAGTCAGAGAACA (for N122); AGTCATCCATGGCCA ATGTGGTCTTCCTCTACAT/GTCATGAATTCATTCTTCAAGTCAGAGAACA (for N200) and AGTCATCCATGGCTCAGCCTCCCGCCCCCCC/GTCATGAATTCTGGGTCGGCATTGATCTGCA (for N279) to add XhoI and NcoI restriction sites and cloning into the vector pRSET A (Invitrogen, Carlsbad, CA). The vectors used to isolate Tat-fusion proteins are derivatives of this vector. Tat-p25 (N122, 200 and 279) were expressed in *E. coli* strain BL21(DE3) pLysS (Novagen, Madison, WI). After sonication in 8M urea, we pelleted the bacterial debris and purified the supernatant by metal-affinity chromatography using a Ni-NTA matrix (Qiagen, Hilden, Germany). We removed salt by gel filtration on Sephadex G-25M (Amersham Biosciences, Uppsala, Sweden) The desalting buffer consisted of Tris 10mM pH6.0; 50% Glycerol, 0,02% Tween-80 and 0,1% Pluronic. We confirmed the identity of proteins by Western blotting (Figure 1). The calculated molecular weights of Tat-p25 D122, D200, and D279 are 27 kDa, 19 kDa, or 28 kDa, respectively. Anti-hemagglutinin (HA) antibodies were purchased from BAbCO (Richmond, CA, USA).

### 2.2. Cell culture and cell death assays

SH-SY5Y cells were plated at a density of 20,000 cells/well of a 96 well dish. Cells were maintained in Dulbecco’s modified Eagle’s medium (PAA, Pasching, Austria) supplemented with 10% fetal bovine serum (PAA), 1 mM L-glutamine, 100 U penicillin/mL, and 100 µg of streptomycin sulfate/mL at 37 °C. 24 h after plating, cells were treated with 10 µM retinoic acid. 

Exactly 24 h after retinoid acid treatment, in all toxicity assays, Tat constructs were applied. Exactly 1 h after protein application, toxic agents were added. 24 h after toxin application, cell survival was assessed by WST viability assay according to the manufacturer’s protocol (Roche, Basel, Switzerland). MnCl_2_ was used at 100 µM concentration. Western blots revealing CDK5 expression after MnCl_2_ treatment were analysed by densitomety (Alpha Innotech Software Version 3.1.2.). Values measured for CDK5 band intensities were normalized by dividing them by the corresponding intensities of the same blot probed with an anti-actin antibody. To induce apoptotic cell death, cells were treated with 400 nM staurosporine for 24 h. 6-OHDA toxicity assays were performed as described [17], with 80 µM 6-OHDA used here. Statistics were performed using the two-tailed student’s t-test.

## 3. Results and Discussion

### 3.1. Mn^2+^ induces Cdk5 expression

**Figure 2:**
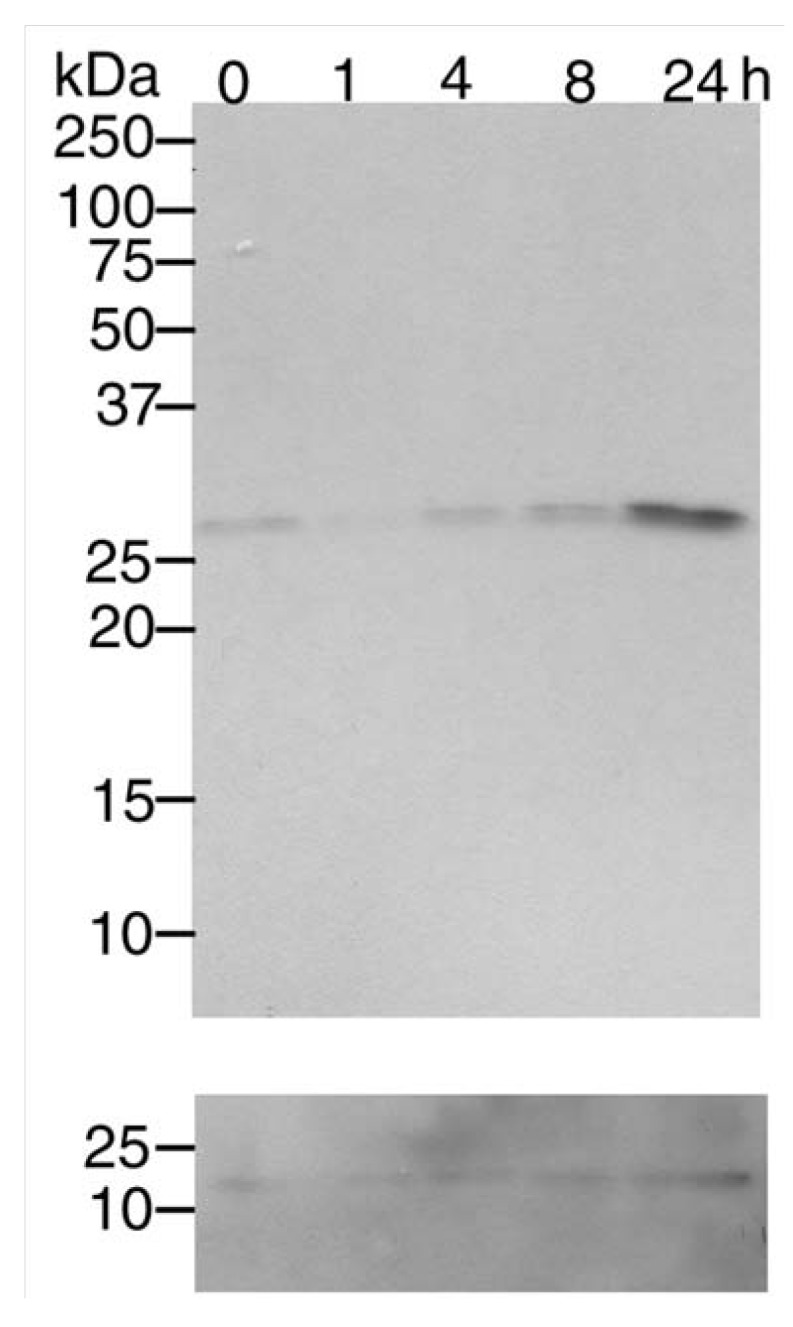
Mn^2+^ treatment induces Cdk5 expression. Differentiated SH-SY5Y neuroblastoma cells were treated with 100µM MnCl_2_ and lysed up to 24 h later as indicated above the lanes. Lysates were examined by Western analysis using an antibody against Cdk5. Anti-Actin staining is shown in the bottom panel.

We treated SH-SY5Y cells with MnCl_2_, as a paradigm for oxidative stress and found that Cdk5 expression was increased up to 24 h after treatment (Figure 2). By densitometric analysis, at 24 h, CDK5 was upregulated 7-fold compared to the 1 h time point. To examine whether cell death induced by oxidative stress was partially mediated by Cdk5 activation, we tested how cell death was modified by dominant-negative p25.

### 3.2. p25 fragments protect SH-SY5Y cells against cell death stimuli

**Figure 3:**
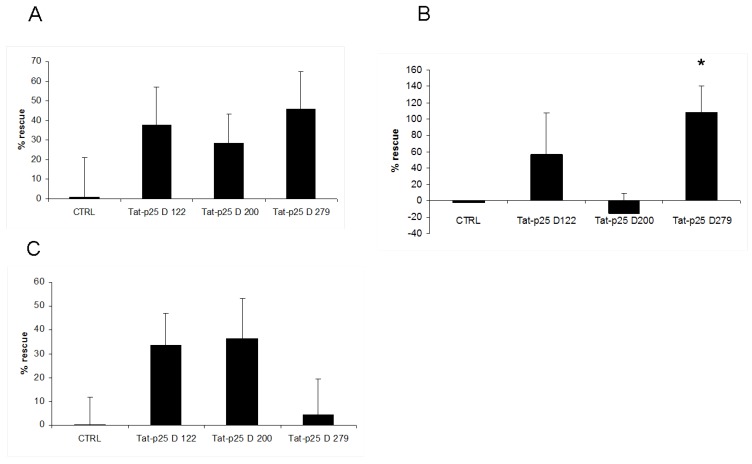
Tat-p25 inhibits apoptosis in neuroblastoma cells. In all paradigms, SH-SY5Y cells were incubated with 100nM Tat peptides before a 24h toxin application. Control cells (CTRL) were treated with a peptide containing only the Tat cell-penetrating peptide without p25. D279 indicates the dominant-negative p25 fragment; D122 and D200 are p25 control fragments. % cells rescued from demise by recombinant protein application after treatment with 100 µM MnCl_2_ (A); 400 nM Staurosporine {B, * indicates significance (p: 0.02)}; or with 80 µM 6-OHDA (C).

Treatment of SH-SY5Y cells with cell-penetrating p25 fragments resulted in statistically not significant trend towards rescue from MnCl_2_-induced cell death. Surprisingly, this was not only observed for the p25 fragment previously reported to act as a dominant-negative [16], but also for other fragments derived from p25 that were reported to bind and still activate, or not to bind to CDK5 (Figure 3a).

We then were interested how the TAT-p25 constructs would influence cell survival in other cell death assays, which have been shown to be influenced by the Cdk5/p25 pathway. Zhang *et al.* were able to show previously, that staurosporine induces cleavage of p35 to p25 with a maximum at 12 and 24 hours, which they suggested to be the cause for staurosporine (STS) induced apoptosis in their paradigm [18]. Moreover, Meuer *et al.* demonstrated CDK5-mediated mitochondrial fragmentation upon staurosporine-treatment of neuronal cells [20]. We observed that a dominant-negative Tat-p25 fragment rescued neuroblastoma cells from cell death induced with 0.4 µM STS (Figure 3b). At higher or lower STS concentrations, we did not observe such an effect (not shown).

To test whether inhibition of Cdk5 would be beneficial in another model for oxidative stress, we tested the p25 mutants in a 6-OHDA toxicity assay; this drug is often applied in models for Parkinson’s disease [20]. None of the applied fragments had a significant effect on cell survival (Figure 3c).

## 4. Conclusions

In our approach we addressed two challenges of therapeutic Cdk5 inhibition: first, we aimed to block its pathological activation, rather than is normal function, by blocking the binding site for p25, and possibly other off-site targets. Secondly, we addressed the problem of drug delivery by using Tat-linked proteins. 

Using *in vitro* studies we addressed different paradigms of cellular stress believed to play a role in neurodegenerative diseases. We applied manganese, which can induce neurodegeneration, including Parkinsonism [21,22]. Manganese is therefore widely used in models for Parkinson’s disease, which includes induction of cell death in SH-SY5Y neuroblastoma cells [23,24,25]. One of its pro-apoptotic actions is mediated by the ERK pathway, by phosphorylating ERK 1 and 2 [26]. It has also been shown to act directly via the NMDA receptor, thereby leading to increased calcium release with subsequent activation of calpains, which cleave p35 to its pathological residuum p25 [27,28]. Our results indicate that p25 fragments, including domains known to bind Cdk5, show a tendency to prevent cell death in this model. Surprisingly, not only a supposedly dominant-negative fragment, but also control p25 fragments that either did not bind CDK5 in previous assays or still did activate it [16] showed a tendency towards a protective effect. We amplified the p25 fragments from whole mouse brains, rather than a bovine source as reported by Poon *et al.* [16]. The cell lines we used originate from human (SH-SH5Y). As we did not test the individual action of each fragment in cell free *in vitro* binding studies or in assays directly assessing the effect on CDK5 enzymatic activity, the previously described functions may not be alienable from bovine to mouse. Moreover, the results obtained using viral transduction and Tat-mediated transduction may not be immediately comparable; the gene expression from different viral constructs may be variable and different from intracellular protein concentrations reached after Tat-mediated transduction [29,30]. Moreover, activation by an *E.coli*-expressed p25 fusion protein may be different from p25 directly expressed and folded in the cell to be examined. In future studies, assays to test the impact of the different Tat-p25 fragments on Cdk5 enzymatic activity could address that issue.

Neither overexpression of a dominant-negative CDK5, nor the CDK5 inhibitors roscovitine and butyrolactone-1 prevented staurosporine-induced cell death [31,32]. Consistent with the results shown here, we have shown earlier that another CDK5 inhibitor protected CSM cells against staurosporine-induced cell death [19]. The discrepancy between those results and the protection we observed might be due to the fact that we applied a dominant-negative form of the CDK5 activator protein p25, rather than directly targeting CDK5, or due to the different cellular system used. The interpretation of the data is further complicated by the fact that staurosporine itself inhibits CDK5 [33].

In a third paradigm, 6-OHDA, which is often used in models for PD, different p25 constructs did not have a significant effect on cell survival; however, there was a trend towards protection with the control constructs. The main apoptotic stimulus by 6-OHDA is believed to be delivered via activation of the ERK pathway [34,35]. Cdk5 in its normal state has been shown to inhibit ERK activation, by phosphorylating MEK1/2. Thus, a dominant-negative p25, as used in our model, may be expected to decrease that inhibition, therefore not protecting against 6-OHDA-induced cell death. On the contrary, some activation of Cdk5 might be expected to alleviate cell death induction.

In conclusion, the effects of virally or cell-penetrating peptide-mediated delivery of proteins may be different. Moreover, the effect of dominant-negative p25 delivery depends on the experimental model examined: For instance, while it blocks staurosporine-induced cell death, it does not protect against 6-OHDA-induced toxicity.
